# A Novel Vision-Based Pose Measurement Method Considering the Refraction of Light

**DOI:** 10.3390/s18124348

**Published:** 2018-12-10

**Authors:** Wei Liu, Xin Ma, Xiao Li, Yi Pan, Fuji Wang, Zhenyuan Jia

**Affiliations:** Key Laboratory for Precision and Non-Traditional Machining Technology of the Ministry of Education, Dalian University of Technology, Dalian 116024, China; maxin2000@mail.dlut.edu.cn (X.M.); 418668164@mail.dlut.edu.cn (Y.P.); wfjsll@dlut.edu.cn (F.W.); jzyxy@dlut.edu.cn (Z.J.)

**Keywords:** triangular vision, pose parameters, machine vision, refraction, wind tunnel test

## Abstract

Nowadays, due to the advantages of non-contact and high-speed, vision-based pose measurements have been widely used for aircraft performance testing in a wind tunnel. However, usually glass ports are used to protect cameras against the high-speed airflow influence, which will lead to a big measurement error. In this paper, to further improve the vision-based pose measurement accuracy, an imaging model which considers the refraction light of the observation window was proposed. In this method, a nonlinear camera calibration model considering the refraction brought by the wind tunnel observation window, was established first. What’s more, a new method for the linear calibration of the normal vector of the glass observation window was presented. Then, combining with the proposed matching method based on coplanarity constraint, the six pose parameters of the falling target could be calculated. Finally, the experimental setup was established to conduct the pose measurement study in the laboratory, and the results satisfied the application requirements. Besides, experiments for verifying the vision measurement accuracy were also performed, and the results indicated that the displacement and angle measurement accuracy approximately increased by 57% and 33.6%, respectively, which showed the high accuracy of the proposed method.

## 1. Introduction

Nowadays, the rapid development of aerospace puts high requirements on the aircraft. The ability of the aircraft to accurately release the internal or external stores (e.g., weapons, auxiliary fuel tanks and bombs) in high-speed airflow directly affects the operation performance [1]. Even worse, the improper separation can threaten the life of the ‘mother machine’ and the pilots. Therefore, to provide a good data reference for the better layout design of the ejection system, as well as the selection of separation parameters, the study of the service performance of an aircraft under simulated airflow environment is necessary. This technology is called the wind tunnel test, among which the separation test is most critical. It allows the evaluation of the performance of the ejection mechanism through the time-varying six-degree-of-freedom parameters (i.e., three position parameters and three angle parameters) of the scaled model falling in the high-speed airflow. However, due to the complex wind tunnel environment (e.g., dark, small test space and high-speed airflow), the traditional contact-based measurement methods (e.g., inertial navigation system, accelerometer and gyroscope) are difficult to use to measure the six parameters of the rolling down target. For the advantages of non-contact, real-time, high-precision and full-field measurement, the vision-based technique has been widely used for detecting both the geometric and motion parameters of a target in complex environments such as undersea, outer planet and wind tunnels [2,3]. Therefore, it is of great significance to study the vision-based method in precisely measuring pose parameters in wind tunnel tests.

The study of vision technology in wind tunnel applications mainly focuses on the measurement of the position, attitude, deformation and vibration parameters of targets. Generally, active or passive luminous markers are installed on the surface of the target as the enhanced features. Two commonly used measurement methods exist, one is to apply a single camera to solve the high-dimensional information of the target by Perspective-n-Point (PNP) algorithms, while the other uses at least two cameras to reconstruct the 3D coordinates of the features based on triangulation. In terms of monocular vision, NASA’s Langley Research Center (LaRC) [4] developed a monocular vision-based system for measuring the angle-of-attack of a model in a wind tunnel test. By processing and analyzing the image sequence of the cooperative markers on the target, the time-varying angle can be calculated. Though this cost-effective and easy-to-operate system has high stability, only pitch angle can be measured, and the position and other two angle information is unavailable. Murray et al. [5] utilized the monocular vision method to deduce the pitch angle and two displacement information of the aircraft in a high-speed wind tunnel. Since only two markers were used, the six pose parameters of the target could not be detected by the proposed system. In 2014, Martinez et al. [6] proposed a pose measurement scheme for a fast flying target in a Mach 4.5 shock tunnel. This method measured the pose parameters by tracking the marker points in the image sequence obtained by a high-speed camera. However, the measurement range was small, and low feature manufacturing accuracy led to high measurement uncertainty. In terms of multi-camera measurement, the NDI of Canada [7] (i.e., the OptotrakTM measurement system with three linear cameras) and the NASA LaRC [8] (two area CCD cameras) achieved the angle measurement in the wind tunnel test. NASA LaRC [9] developed the videogrammetric model deformation (VMD) measurement system, combining two cameras with the reflective markers, the torsion, bending, deformation and angle-of-attack parameters of the object, which can be accurately calculated. This system has been well applied in various low, high and ultra-high speed wind tunnel tests in the United States, Canada, etc. However, the maximum measurement frequency of 250 Hz made it unable to measure pose information of fast moving targets. Similarly, Sant et al. [10] from the French national aerospace research center (ONERA) developed a two-camera based wing model deformation measurement system (MDM). Though the wing distortion measurement accuracy was better than 0.05°, the low measurement frequency (10 Hz) limited its application in high dynamic measurement. In addition, research on both monocular and binocular vision has been conducted in our previous works. Liu et al. [2] presented a pose measurement scheme using the self-luminous color markers as the enhanced feature. The binocular vision-based measurement experiments were conducted for the high-speed isolates, and high-accuracy results were obtained. Additionally, together with a high-intensity light source and retro-reflective targets, in [11], we used two cameras to measure the pose information of a small and moving target. Furthermore, a flexible monocular vision-based measurement method and system [12] has been proposed and applied in a wind tunnel test. To increase the flexibility of the vision system for different wind tunnel applications, an optical lens that enables the fast adjustment of the focal length is included in the measurement system. Many scholars have investigated different kinds of vision-based innovations in calculating the movement parameters of the flying targets in wind tunnels, and good results have been achieved. However, in practical measurements, cameras should observe the dropping targets through the glass observation window to protect the vision system from the influence of the high-speed airflow. Consequently, the introduced light refraction reduces the measurement accuracy, especially for measurement in high-speed airflow and relatively thick glass window conditions. However, for the existing vision-based methods, the impact of the window glass thickness and the high-speed airflow is seldom taken into consideration.

Here, aiming at further improving the vision-based measurement accuracy, we propose an improved metrology for estimating the pose information of a falling target in wind tunnel tests, considering the refraction caused by the observation window and high-speed airflow. To this end, Section 2 presents the photography model based on refraction geometry; in Section 3 the corresponding camera calibration method is described; Section 4 details the scheme for high signal-to-noise ratio (SNR) image acquisition of the moving target, while in Section 5, the image processing for detecting the six movement parameters is introduced; Section 6 presents experimental results in the laboratory and Section 7 gives the conclusion of this paper.

## 2. Photography Model under Wind Tunnel Environment

**Figure 1:**
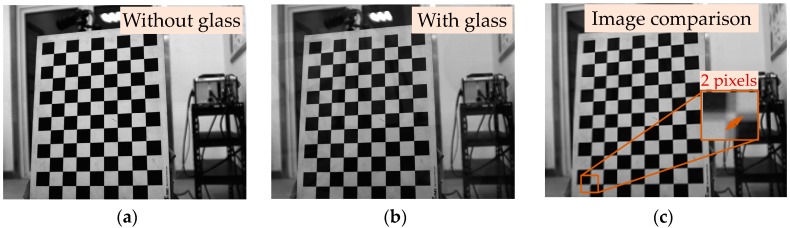
Corner shift caused by refraction of the light: (**a**) Without glass; (**b**) with glass; (**c**) corner shift.

In practical applications, the large thick observation window should be embedded in the wall to protect the vision measurement system from high-speed air flow. However, the glass medium has a different refractive index from that of air, which will inevitably introduce the light refraction at the glass-air interface. As shown in Figure 1, images of a calibration target with (Figure 1a) and without (Figure 1b) a 1 cm thick optical glass were acquired by the camera. The comparison results (Figure 1c) indicated that the corner shift was up to 2 pixels, as a result, this positive thickness-related pixel shift greatly decreased the measurement accuracy of the optical system. Therefore, for the vision measurement under complex wind tunnel environments, the influence of optical thick glass should be taken into consideration in the imaging model.

As illustrated in Equation (1), for a conventional camera imaging model, it describes the one-to-one mapping between a space control point $P_{w}\left(\begin{array}{ccc} X_{w} & Y_{w} & Z_{w} \end{array}\right)$ and the corresponding two-dimensional pixel projection $p\left(\begin{array}{cc} u & v \end{array}\right)$:

(1) $$Z_{c}\left[\begin{array}{c} u\\ v\\ 1 \end{array}\right]=\underset{K}{\underbrace{\left[\begin{array}{ccc} \frac{1}{d_{x}} & 0 & c_{x}\\ 0 & \frac{1}{d_{y}} & c_{y}\\ 0 & 0 & 1 \end{array}\right]\left[\begin{array}{cccc} f & 0 & 0 & 0\\ 0 & f & 0 & 0\\ 0 & 0 & 1 & 0 \end{array}\right]}}\left[\begin{array}{cc} R & T\\ 0^{T} & 1 \end{array}\right]\left[\begin{array}{c} X_{w}\\ Y_{w}\\ Z_{w}\\ 1 \end{array}\right]$$

where f denotes the lens focal length; the principal point $\left(\begin{array}{cc} c_{x} & c_{y} \end{array}\right)$ with respect to the image coordinate frame is expressed in pixel; $d_{x}\times d_{y}$ describes the dimension of one photosensitive element on the sensor array; K is the intrinsic matrix, while the extrinsic matrix $\left[\begin{array}{cc} R & T \end{array}\right]$ represents the transformation information between world and camera coordinate frames. However, when the glass media is introduced, the traditional pinhole model gives an improper description of imaging mapping, as a result, vision measurement accuracy would be reduced [13].

**Figure 2:**
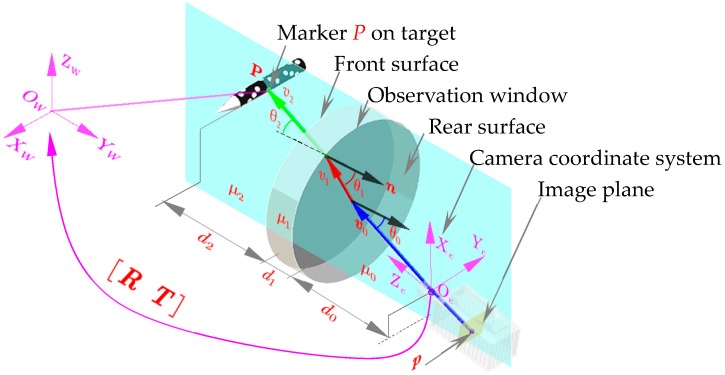
Principle of the photography model in wind tunnel.

Thus, an imaging model including the refraction effect of the medium was applied in this paper. As shown in Figure 2, the outgoing rays were classified into three segments according to the disparity of media: The first segment formed by a pixel point and principal center from the image plane to the rear surface, the second inside the observation window, and the third from the front surface to the target. $v_{0}$, $v_{1}$ and $v_{2}$ indicate the direction vector of each segment, respectively; $\mu_{0}$, $\mu_{1}$, $\mu_{2}$ depict the refractive index of air, observation glass and high speed airflow, while the thicknesses of each medium layer was recorded as $d_{0}$, $d_{1}$ and $d_{2}$; the normal vector of observation window is given by n. From Snell’s law, $u_{i}\cdot \sin\theta_{i}=u_{i+1}\cdot \sin\theta_{i+1}$, where $\theta_{i}$ denotes the angle formed by $v_{i}$ and n. The expression can be further vectorized referring to [14]:

(2) $$v_{i+1}=a_{i+1}\cdot v_{i}+b_{i+1}\cdot n$$

where $a_{i+1}=u_{i}/u_{i+1}$; $b_{i+1}=\frac{-u_{i}\cdot v_{i}^{T}\cdot n-\sqrt{u_{i}^{2}\cdot \left(v_{i}^{T}\cdot n\right)-\left(u_{i}^{2}-u_{i+1}^{2}\right)\cdot v_{i}^{T}\cdot v_{i}}}{u_{i+1}}$; $v_{0}$ can be calculated according to the intrinsic matrix K:

(3) $$v_{0}=\left[\begin{array}{c} \frac{u\cdot d_{x}}{\sqrt{u^{2}\cdot d_{x}^{2}+v^{2}\cdot d_{y}^{2}+f^{2}}}\\ \frac{v\cdot d_{y}}{\sqrt{u^{2}\cdot d_{x}^{2}+v^{2}\cdot d_{y}^{2}+f^{2}}}\\ \frac{f}{\sqrt{u^{2}\cdot d_{x}^{2}+v^{2}\cdot d_{y}^{2}+f^{2}}} \end{array}\right]$$

Then, according to the principle of refraction geometry, the spatial information of an object point P can be expressed as:

(4) $$P=-\frac{d_{0}\cdot v_{0}}{v_{0}^{T}\cdot n}-\frac{d_{1}\cdot v_{1}}{v_{1}^{T}\cdot n}-\frac{d_{2}\cdot v_{2}}{v_{2}^{T}\cdot n}$$

As can be seen from the established imaging model, in addition to the intrinsic matrix K and extrinsic matrix (R,T) in the traditional pinhole camera model (see Equation (1)), the established imaging model also contains the normal vector n and the thickness of the each medium layer $d_{0}$, $d_{1}$, $d_{2}$. In order to calibrate the above imaging parameters, priori data were required. In our study, the known priori data included refractive indices and spatial object point P on the calibration target. The former included the refractive index of the air $\mu_{0}$, optical window $\mu_{1}$ and the constant airflow $\mu_{2}$ in the wind tunnel. While the latter spatial feature points P were calibrated by high-precision photogrammetric device using resection. It should be noted that the image projection p of feature point P on the image plane can be located by image-processing.

## 3. Calibration Method

**Figure 3:**
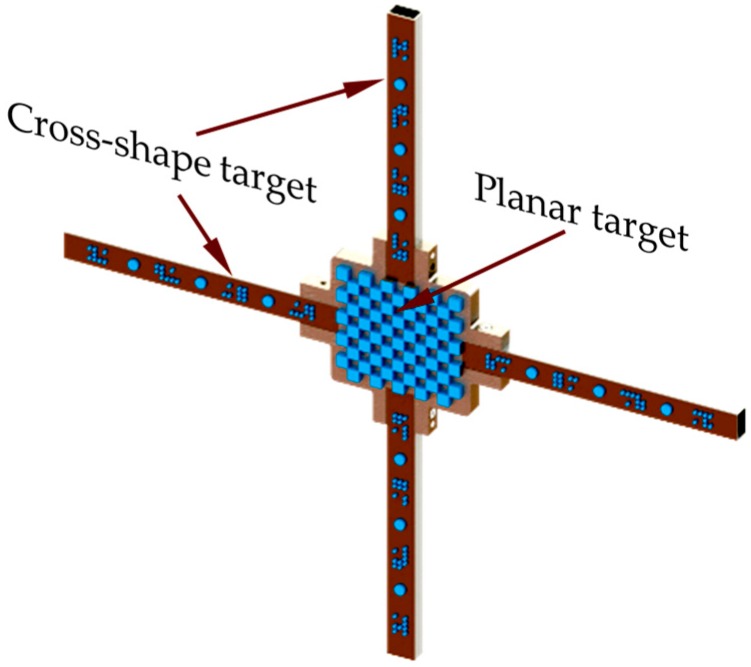
Compound target used for camera calibration.

Based on certain captured images of multi-pose calibration target, by solving the co-linearity equations, camera calibration allowed for the estimation of intrinsic parameters, which characterized the properties of the optical system, as well as the extrinsic parameters. However, for large-scale space calibration in wind tunnels, the use of conventional planar chessboard is expensive and inconvenient. In this paper, a convenient compound target was applied to address the problem. As illustrated in Figure 3, the target consists of an outer cross-shape frame and a central planar target. In this paper, to further improve the vision-based pose measurement accuracy, an imaging model, which considers the refraction light of the observation window was proposed. As illustrated in Section 2, the large pixel shift caused by the refraction effect of glass observation window greatly decreased the measurement accuracy of the optical system. Therefore, parameters (i.e., thickness $d_{1}$ and normal vector of the window n) that can well reflect the refractive effect are the major contributors to the vision measurement uncertainty, which needs to be calibrated accurately. If we apply optimization algorithm to calibrate the overall system, parameters (intrinsic matrix K, extrinsic matrix (R,T) representing the lens and the camera properties will cause a coupling effect on the solution of n. That is to say, though the combination of multiple parameter values can achieve a relatively small cost function, the parameter n of the calibrated optical glass was not optimal. In view of this, we performed a step-by-step calibration method to accurately calculate the parameters in the proposed imaging model: (1) Calculate the internal matrices of the two cameras using Zhang’s method [15] without installing the observation widow; (2) determine the normal vector n of the observation window; (3) calculate the external parameters and thickness of the medium layers.

### 3.1. Calibration Method for the Normal Vector of the Observation Window

There are many methods for measuring the thickness and the normal vector of the observation window glass [14,16]. However, several parameters interact with one another when solving the equation, which decreases the solution accuracy and robustness.

**Figure 4:**
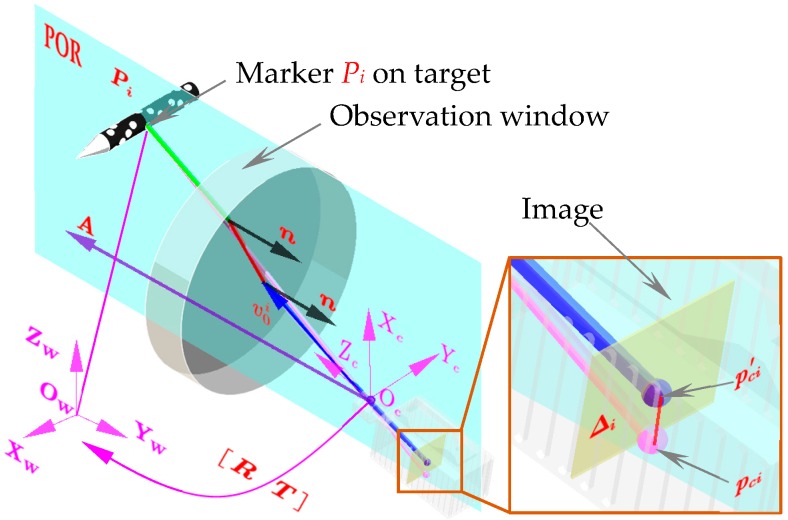
Principle of the parallel superposition method.

In this paper, the measurement method for calculating the normal vector of the observation window using the image deviation was proposed and the principle is shown in Figure 4. A is the axis which is parallel to the normal vector and goes through the optical center $O_{c}$; $P_{i}$ is the ith object point in the measurement space. $p_{ci}$ and ${p^{\prime }}_{ci}$ are the pixel point that $P_{i}$ forms on the image coordinate frame before and after installing the observation window, respectively. The image deviation caused by the installation of the observation window is thus defined as $\Delta_{i}=p_{ci}-{p^{\prime }}_{ci}$. The normal vector n of the observation window is calculated linearly by the distortion vector, which occurred in the picture when the observation window was installed. Specific methods are as follows.

As described in Figure 4, the flat refraction system with two-layer mediums for a corresponding axial camera was first proposed. Let Π be the plane of refraction (POR) formed by the axis and an outgoing ray, hence, the normal vector n is in the plane Π. Judging by Snell’s law, the incident light, n and the refracted light are coplanar at an arbitrary boundary when refraction occurs. Thus, the whole refracted camera ray should locate on Π, meanwhile the last refracted light and the axis A are intersecting because both of them are located on the Π. Therefore, all outgoing rays are coplanar with A, and the system is axially distributed. Hence, the spatial point RP+T transformed to the world coordinate frame should also be located in Π. Accordingly, for each spatial point, the coplanarity constraint conforms to the following expression:

(5) $${\left(RP+T\right)}^{T}\cdot \left(n\times v_{0}\right)=0$$

Since the observation window is added, the obtained image will be distorted. However, the distorted pixels and the distortion vectors Δ still remain in the plane of refraction (POR). Therefore, for each spatial object point, Δ and n satisfy:

(6) $$n\cdot \left(\Delta \times v_{0}\right)=0$$

where $\Delta \times v_{0}$ is the normal vector of POR. It should be noted that when the system parameters such as thicknesses $d_{i}$, refractive indices $\mu_{i}$ and the number of layers are changed, the coplanarity constraint remains unchangeable. It is only relevant to the normal vector of the window n and the distortion vectors Δ. With Equation (6), the normal vector of the window can be easily deduced alone in a linear way.

### 3.2. Calculation Method for the External Parameters of the Camera

In this section, the calculation method for external parameters of the camera is introduced. Firstly, the columns of a matrix R are stacked to form the vector R(:), and the kronecker product is denoted by ⊗. Then, the coplanarity constraint (Equation (5)) can be re-written as:

(7) $${\left(n\times v_{0}\right)}^{T}\cdot \left(RP+T\right)=0$$

In which we have 6 degrees of freedom: 3 for rotation and 3 for translation. Then we can get a linear system:

(8) $$\underset{B}{\underbrace{\left[\begin{array}{cc} n\times v_{0}^{1}⊗P_{1} & n\times v_{0}^{1}\\ \vdots & \vdots \\ n\times v_{0}^{12}⊗P_{12} & n\times v_{0}^{1} \end{array}\right]}}\cdot \left[\begin{array}{c} R\left(:\right)\\ T \end{array}\right]=0$$

where B is a 12×12 matrix whose rank is 12. By solving this equation, we can obtain R and T. Then, in order to further calculate the thickness of layers, we induced another constraint:

(9) $$v_{n}\times \left(RP+T-q_{n}\right)=0$$

which demonstrates that the last ray $v_{n}$ is parallel to the line connecting the refracted points $q_{n}$ to the spatial point RP+T. $q_{n}$ and can be described as $q_{n}=\Sigma_{i=0}^{n-1}-d_{i}\cdot v_{i}/\mu_{i}$. By substituting, we get the following equation:

(10) $$v_{n}\times \left[\begin{array}{cccc} \frac{v_{0}}{\mu_{0}} & \frac{v_{1}}{\mu_{1}} & \cdots & \frac{v_{n-1}}{\mu_{n-1}} \end{array}\right]\left[\begin{array}{c} d_{0}\\ d_{1}\\ \vdots \\ d_{n-1} \end{array}\right]=-v_{n}\times \left(RP+T\right)$$

After calculating this equation, we can obtain the value of $d_{0}$, $d_{1}$ and $d_{2}$.

## 4. High Signal-to-Noise Ratio Image Acquisition of the Falling Target

In a practical measurement, the small-sized target was dropped from the scaled aircraft model by the ejection mechanism at high speed, At the same time, two high-speed cameras were synchronously triggered to collect the motion information of the small rolling down targets through a small observation window. Thus, a clear image sequence of the fast moving target in such a complex environment was difficult to be acquired [17].

**Figure 5:**
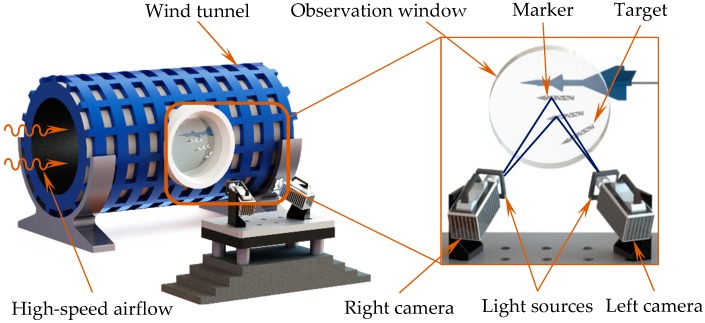
The image acquisition method.

**Figure 6:**
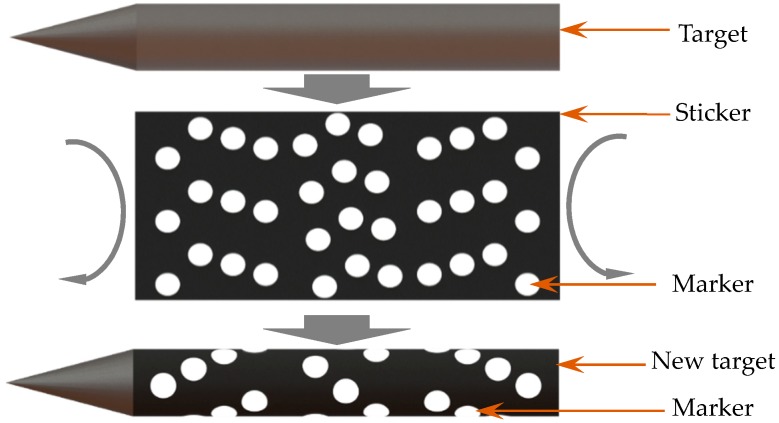
The measurement target.

Therefore, a method for image acquisition was adopted based on reflecting markers, as shown in Figure 5. Furthermore, for the purpose of ensuring the aerodynamic performance of the scaled target, the thickness of each marker should be less than 30 μm. However, it was difficult to guarantee the measurement accuracy by using common markers (160 μm). To effectively improve the aforementioned measurement accuracy, a fabrication process technology, which prints the retro-reflection markers without protuberant was presented to satisfy the measurement requirements of a fast falling target under high-speed airflow, which also guaranteed the relative positions of the markers and had no side-effect for measurement. The markers were printed smoothly onto a sticker, and then the sticker was attached to the target as shown in Figure 6.

**Figure 7:**
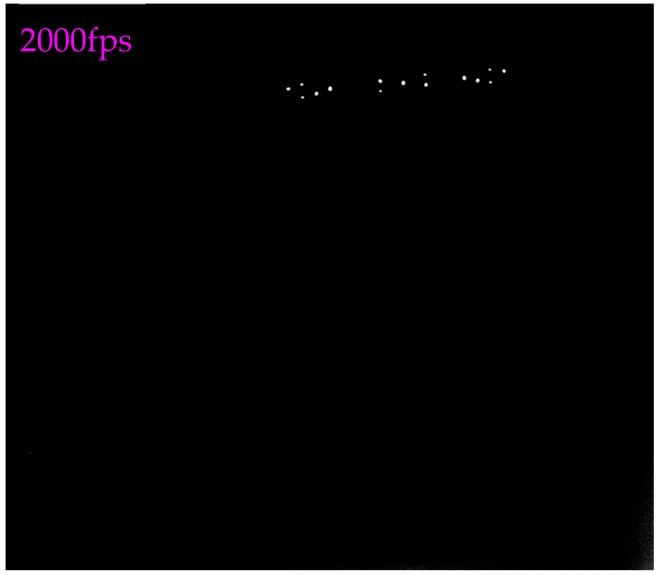
Image of the falling target captured by the proposed image collection method (at 2000 fps).

With this method, the thickness of each marker could be controlled to 20 μm, meanwhile the corresponding positioning accuracy could be guaranteed. To further validate the quality of the image captured by the proposed scheme, the image sequence of the markers on the high-speed falling target were acquired by the two cameras with 2000 fps in the wind tunnel test. Figure 7 displays one target’s image shooting by the left camera. The results indicated that the SNR and brightness of the acquired image in complex measurement conditions is excellent compared to the measurement method in reference [17].

## 5. Image Processing

**Figure 8:**
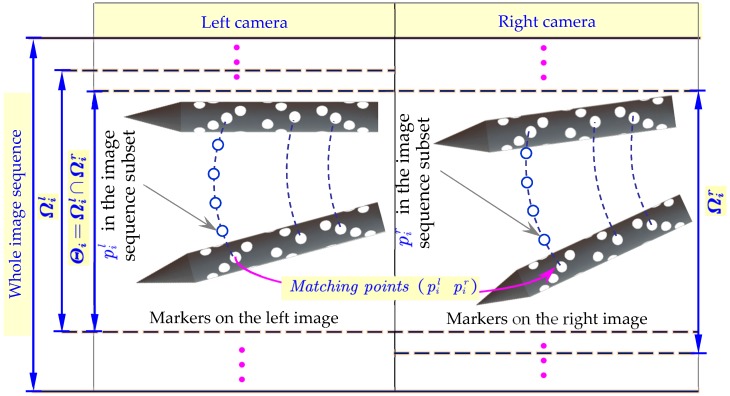
Principle of the matching method.

As shown in Figure 8, in this paper, a binocular vision based method was proposed for calculating the pose parameters of the falling target in a wind tunnel test. The most critical step in reconstructing the 3D coordinates of a spatial point using binocular vision is to match feature points in the two view images. For this intension, firstly, the two cameras were triggered synchronously by the application in the image acquisition software (PFV) to ensure the accurate correspondence between the two view images. Then, as mentioned in Section 4, ultra-thin markers distributed on the target surface were designed to characterize the six-dimensional motion information of the target. To improve the feature matching efficiency, before matching, all the markers $p_{i}$ appearing in the image sequence were tracked referring to the method in [18,19,20]. Additionally, the continuous left $\Omega_{i}^{l}=\left[\begin{array}{cc} {\mathrm{lower}}_{i}^{l} & {\mathrm{higher}}_{i}^{l} \end{array}\right]$ and right $\Omega_{i}^{r}=\left[\begin{array}{cc} {\mathrm{lower}}_{i}^{r} & {\mathrm{higher}}_{i}^{r} \end{array}\right]$ image sequence subsets to which each marker $p_{i}$ belonged could be obtained. Thereafter, feature matching was performed. Due to the multi-layer refraction caused by the observation window, the matching points in the two view images will not be in agreement with the conventional limited constraints [20,21,22], and traditional matching methods will decrease the feature matching accuracy of markers, even affecting the final measurement accuracy.

**Figure 9:**
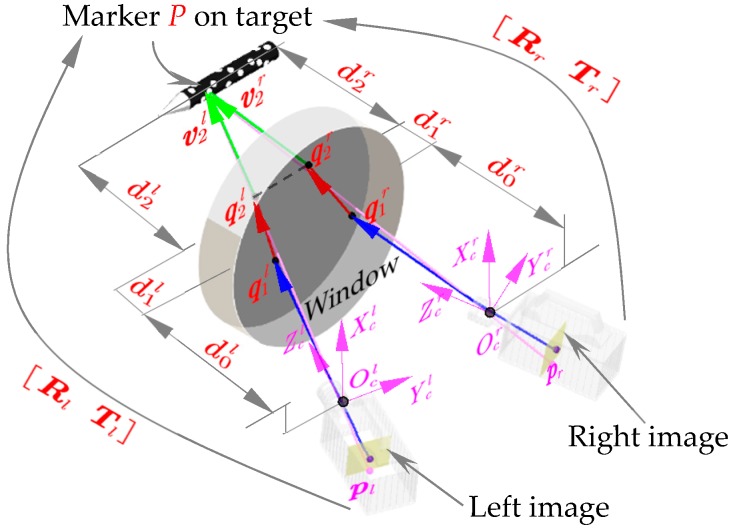
Schematic diagram of image matching constraint.

Therefore, a matching method in virtue of the multi-layer refraction coplanar constraints was proposed to solve the practical measurement problem. As shown in Figure 9, $v_{2}^{l}$ and $v_{2}^{r}$ intersected at P, so $q_{2}^{l}$, $q_{2}^{r}$ and P were in the same plane, then the matching equations $F_{3}\left(p_{l},p_{r}\right)$ between $p_{l}\left(u_{l},v_{l}\right)$ and $p_{r}\left(u_{r},v_{r}\right)$ could be written as:

(11) $$F_{3}\left(p_{l},p_{r}\right)=\left(R_{lr}\cdot q_{2}^{r}+T_{lr}-q_{2}^{l}\right)\left[\left(R_{lr}\cdot v_{2}^{r}\right)\times v_{2}^{l}\right]=0$$

where $\left[\begin{array}{cc} R_{l} & T_{l} \end{array}\right]$ and $\left[\begin{array}{cc} R_{r} & T_{r} \end{array}\right]$ represent the calibrated extrinsic parameters of the two cameras with respect to the world coordinate frame, respectively. Then for each frame of the left image sequence, the marker $p_{i}^{r}$ in the corresponding right image, which matched $p_{i}^{l}$ in the left image was found by using Equation (11), and we got $\left(\begin{array}{cc} p_{i}^{l} & p_{i}^{r} \end{array}\right)$. On this basis, we calculated the number of images that marker $p_{i}$ appeared simultaneously in the two view image sequences $\Theta_{i}$, $\Theta_{i}=\Omega_{i}^{l}\cap \Omega_{i}^{r}$, then we believe that image point $p_{i}^{l}$ and $p_{i}^{r}$ are matched in subset $\Theta_{i}$ of both image sequences. That is to say, there was no need to judge the matching relation of $p_{i}$ using Equation (11) in the subsequent $\Theta_{i}-1$ number of images, and the image matching efficiency was improved. Finally, the fast matching of all feature points could be completed by repeating the steps above.

After feature matching, the spatial coordinates of the markers could be constructed by triangulation. Thereafter, the pose information of the moving target was achieved with the aid of the aforementioned markers layout [11]. More specifically, to characterize the coordinate system transformation, we set the initial coordinate frame at the first image, which was denoted by ICF; while the local coordinate frame LCF was fixed on the other image frames, the origins of two coordinate frames located on the centroid of the target.

**Figure 10:**
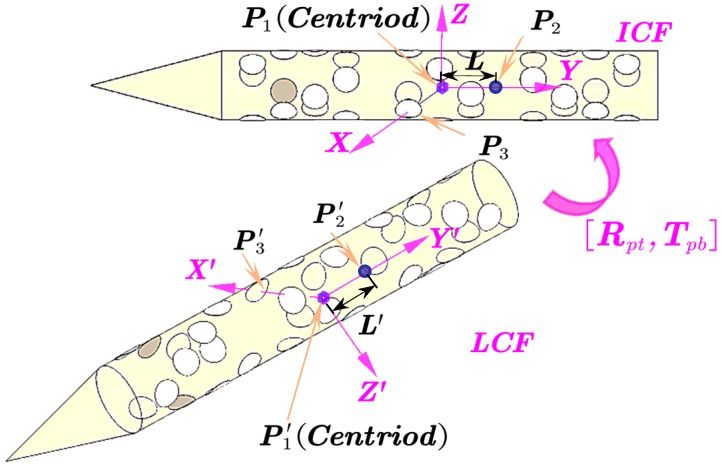
Schematic of principle for absolute pose solution.

As shown in Figure 10, centroid $P_{1}$ was the origin of the LCF after fitting the axis of the target [16,23]. Then, we considered this line as the Y axis of the coordinate frame. The second constant point $P_{2}$ was defined on the Y axis along which the distance from the point $P_{2}$ to original point $P_{1}$ was L. The optimum point $P_{3}$ on the surface of the target represented the third point. Then the rotation matrix $R_{pt}$ and translation matrix $T_{pb}$ could be calculated by the absolute position of the corresponding three points. $R_{pt}$ could be obtained by the coordinate system rotating around a spatial unit vector for a fixed angle, or rotating around its axis in order. In this paper, the latter method was utilized to obtain rotation and translation parameters of the target.

The relationship between the point $p={\left(\begin{array}{ccc} x & y & z \end{array}\right)}^{T}$ in ICF and the corresponding $p^{\prime }={\left(\begin{array}{ccc} x^{\prime } & y^{\prime } & z^{\prime } \end{array}\right)}^{T}$ in LCF can be written by:

(12) $$\left[\begin{array}{c} x^{\prime }\\ y^{\prime }\\ z^{\prime }\\ 1 \end{array}\right]=\left[\begin{array}{cc} R_{pt} & T_{pb}\\ 0^{T} & 1 \end{array}\right]\left[\begin{array}{c} x\\ y\\ z\\ 1 \end{array}\right]$$

where $R_{pt}$ and $T_{pb}$ describe the coordinate system transformation between ICF and LCF.

And

(13) $$\begin{array}{l} R_{pt}=R_{\theta_{Y}}\cdot R_{\theta_{X}}\cdot R_{\theta_{Z}}\\ =\left[\begin{array}{ccc} \cos\left(\theta_{Y}\right)\cos\left(\theta_{Z}\right)-\sin\left(\theta_{Y}\right)\sin\left(\theta_{X}\right)\sin\left(\theta_{Z}\right) & \cos\left(\theta_{Y}\right)\sin\left(\theta_{Z}\right)+\sin\left(\theta_{Y}\right)\sin\left(\theta_{X}\right)\cos\left(\theta_{Z}\right) & -\sin\left(\theta_{Y}\right)\cos\left(\theta_{X}\right)\\ -\cos\left(\theta_{X}\right)\sin\left(\theta_{Z}\right) & \cos\left(\theta_{X}\right)\cos\left(\theta_{Z}\right) & \sin\left(\theta_{X}\right)\\ \sin\left(\theta_{Y}\right)\cos\left(\theta_{Z}\right)+\cos\left(\theta_{Y}\right)\sin\left(\theta_{X}\right)\sin\left(\theta_{Z}\right) & \sin\left(\theta_{Y}\right)\sin\left(\theta_{Z}\right)-\cos\left(\theta_{Y}\right)\sin\left(\theta_{X}\right)\cos\left(\theta_{Z}\right) & \cos\left(\theta_{Y}\right)\cos\left(\theta_{X}\right) \end{array}\right] \end{array}$$

where $-\theta_{Z}$, $-\theta_{X}$ and $-\theta_{Y}$ are defined as the respective yaw, pitch and roll angles in the world coordinate frame. Based on the selected points, the target pose information in the movement-time history could be obtained.

## 6. Experiment and Results

### 6.1. Vision-Based Measurement System

**Figure 11:**
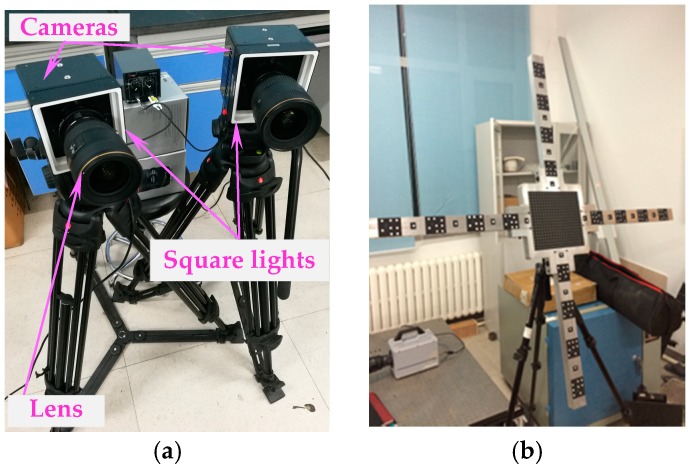
Measurement system and compound target used for cameras’ calibration: (**a**) Binocular vision measurement system; (**b**) real compound target.

The established binocular vision-based measurement system is described in Figure 11. It included two high-speed FASTCAM Mini cameras from Photon Inc. (Tokyo, Japan), the corresponding two wide-angle Nikon 17-35 lenses from Nikon Inc. (Tokyo, Japan) and two low-angle square lights from CCS China Inc. (Shenzhen, China), as well as a graphic workstation. Each lens was surrounded by a square light, which was used to ensure the high uniform illumination in the wind tunnel. Images were captured by two cameras with a resolution of 1024×1280 pixels at a sample frequency of 2000 fps, which guaranteed the image quality and the detail resolution of the target.

### 6.2. Accuracy Validation Experiments

**Figure 12:**
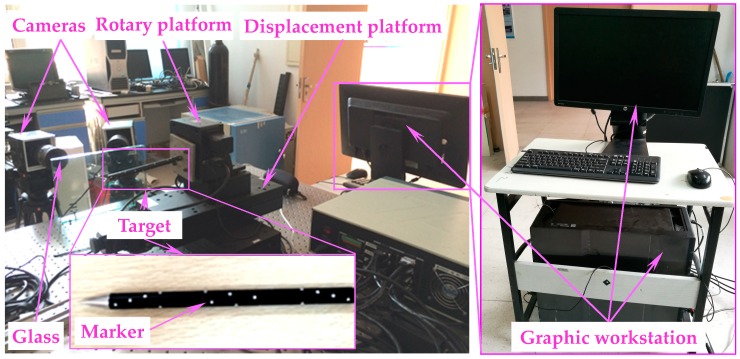
Experimental facilities for verifying the accuracy of the proposed method.

The traditional pinhole camera model allows the direct mapping of 3D spatial points to 2D image projections through the optical imaging system. However, after the optical glass is introduced into the light-path, the vision measurement uncertainty is increased. To address the problem, we proposed a pose measurement method, which considered the refraction of light. Before applying the proposed method in a practical application, the accuracy of the proposed method in measuring pose parameters of the falling target was testified and then compared with that of the conventional method in the laboratory. To conduct the experiment, firstly, the target was installed on the high-accuracy electronic control platform, and the optical glass with thickness of 30 mm was placed between the target and the imaging system (see Figure 12 below). Then, camera models considering (i.e., the camera model proposed in this paper) and not considering (the conventional pinhole model) the refraction effect of the optical glass were calibrated, respectively. Thereafter, for each measurement model, the electronic control stage drove the target to individually move several known positions in the six-degree-of-freedom direction (X, Y, Z, pitch, yaw and roll). The measurement field was 650 mm × 650 mm.

**Figure 13:**
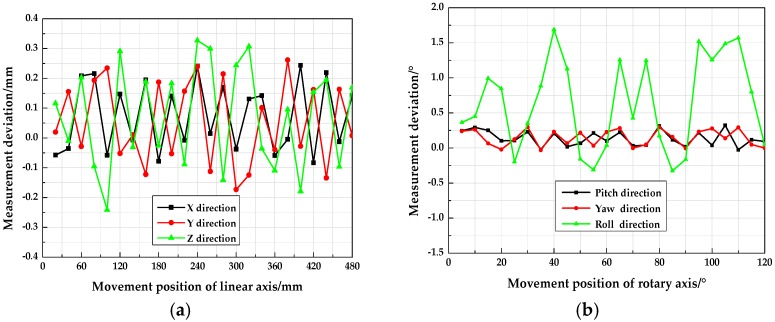
Position and attitude accuracy measurement with a pinhole model: (**a**) Displacement measurement deviation in three linear axes; (**b**) measurement deviation of the pitch, yaw and roll angle.

**Figure 14:**
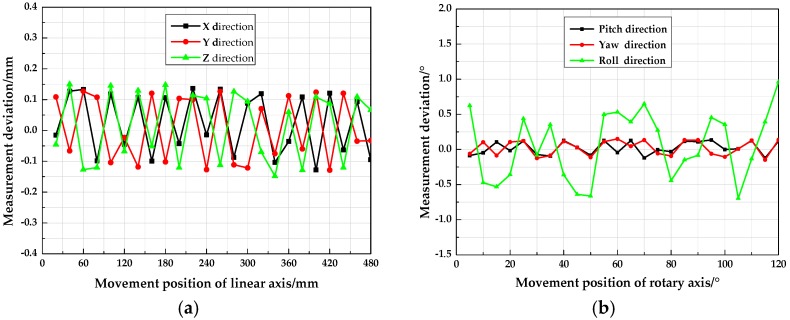
Position and angle measurement accuracy with the refraction model: (**a**) Displacement measurement deviation in three linear axes; (**b**) measurement deviation of the pitch, yaw and roll angle.

As shown in Figure 12, the high accuracy electronic control platform consisted of both displacement and rotary axes. The position accuracy of 0.001 mm enabled the electronic control platform to control the movement of the target with precise known data. In terms of the three linear axes (i.e., *X*-, *Y*- and *Z*-axis), the position measurement accuracy for each axis was verified by individually moving the target to 24 positions at 20 mm intervals. While, the angle measurement accuracy for each rotary axis was validated by separately rotating the target to 12 positions at 5° intervals around the axis. Finally, the accuracy of the two measurement models in measuring the six pose parameters of the target was verified by the constructed distance and angular errors of two adjacent positions. Figure 13 shows the position and attitude accuracy calculated by a conventional pinhole model as referred to in [11]. Figure 14 illustrates the position and attitude accuracy measured with the method proposed in this paper.

As shown in Figure 13, with the pinhole model, the mean square error (MSE) of the displacement measurement in the *X*-, *Y*- and *Z*-axis direction was 0.216 mm, 0.221 mm and 0.215 mm, respectively; while, the MSE of pitch, yaw and roll angle measurement were 0.183°, 0.171° and 0.826°. It should be highlighted that the imperfection of the traditional imaging model and camera calibration method were the major contributors to error. As shown in Figure 14, with the refraction model, the MSE of the displacement measurement in the corresponding *X*-, *Y*- and *Z*-axis direction were 0.091 mm, 0.092 mm and 0.090 mm. While, the MSE of the pitch angle measurement was 0.090°, with the yaw angle measurement error of 0.093°, and the roll angle measurement error of 0.548°. By using the proposed method considering the refraction model, the displacement and angle measurement accuracy approximately increased by 57% and 33.6%, respectively, which showed the high accuracy of the measurement method.

### 6.3. Vision Measurement Experiment

**Figure 15:**
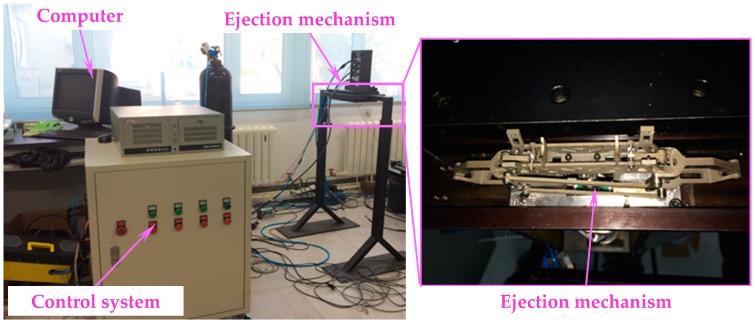
Ejection system.

In the laboratory, an experimental setup consisting of both the vision measurement system and the separation system was established to simulate the wind tunnel test conditions. In a practical measurement, the target is ejected by the ejection mechanism at a specified speed (Figure 15). Simultaneously, the calibrated binocular vision system collects the image sequence of the target at 2000 fps with a large field of view of 1 m × 1 m through a 30 mm thick glass. Then, the time-varying pose parameters can be calculated by image analysis of the markers’ positions in an image sequence.

**Figure 16:**
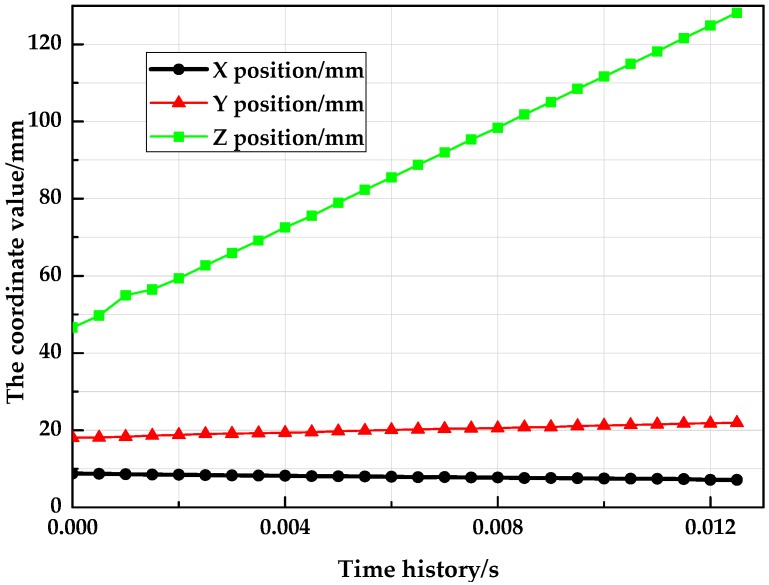
Results of the position measurement.

**Figure 17:**
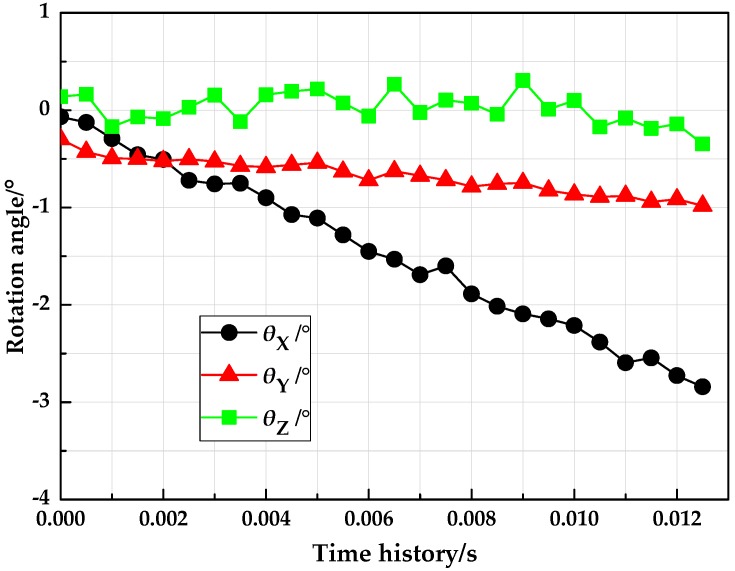
Results of the attitude measurement.

Since the target is released by the ejection mechanism, the path formed in the time history is similar to free-fall trajectory. The measured position information of the target is illustrated in Figure 16, while Figure 17 shows the angle measurement results. It can be illustrated from the plot data that the trajectory of the moving target was in line with the expected form of motion, additionally, the measured velocity was in full accordance with the preset value, which indicated that this system could estimate the six pose parameters of the fast falling target.

## 7. Conclusions

Aimed at reducing the pinhole model-based vision measurement uncertainty caused by the glass observation window, we furthered the study, and proposed an improved vision-based pose measurement method, which considers the refraction of light. The main contribution of this paper was to include the light-path changes caused by optical refraction into the traditional imaging model, and to calibrate the model in a multi-step way to remove the coupling effect of imaging parameters. Then, in combination with the designed ultra-thin markers, that ensures the aerodynamic shape of the model, the six-degree-of-freedom information can be constructed after matching the markers using the multi-layer refraction coplanar constraints. Both accuracy verification and pose measurement experiments were carried out in the laboratory. The verification results indicated that the displacement and angle measurement accuracy approximately increased by 57% and 33.6%, respectively. This proposed a high-accuracy pose measurement method that could provide a good reference for improving the data accuracy of other relevant vision-based wind tunnel special experiments (e.g., deformation measurement test, free flight experiment and vibration measurement test). Our next step is to consider the airflow influence in a wind tunnel test with time-varying wind speed to further enhance the vision measurement accuracy.
